# Aging and aging-associated diseases: a microRNA-based endocrine regulation hypothesis

**DOI:** 10.18632/aging.101612

**Published:** 2018-10-29

**Authors:** Samuil Umansky

**Affiliations:** 1 DiamiR Biosciences, Monmouth Junction, NJ 08852 USA

**Keywords:** geroscience, miRNA hormones, pituitary gland, sex-dependent differences, degeneration and carcinogenesis, Down syndrome

## Abstract

Although there are numerous hypotheses explaining the nature of aging and associated processes, two concepts are dominant: (i) aging is a result of cell-autonomous processes, such as the accumulation of DNA mutations, aberrant methylations, protein defects, and shortening of telomeres, leading to either inhibition of cellular proliferation and death of non-dividing terminally differentiated cells or tumor development; (ii) aging is a result of a central program that is switched on at a specific stage of organismic development. The microRNA-based endocrine regulation hypothesis combines the two above concepts by proposing central regulation of cell death occurrences via hypothalamus-pituitary gland (PG)-secreted miRNA hormones, the expression and/or secretion of which are regulated by sex hormones. This hypothesis explains such well-known phenomena as inverse comorbidity of either cancer or Alzheimer’s (AD) and other neurodegenerative diseases; higher AD morbidity and lower frequency of many common types of cancer in women vs. men; higher risk of early AD and lower risk of cancer in subjects with Down syndrome; longer life expectancy in women vs. men and much lower sex-dependent differences, if any, in other mammals; increased lifespans due to hypophysectomy or PG hypofunction; and parabiotic effects of blood or plasma transfusions between young and old animals.

## Introduction

After considerable success in fighting infections and the significant increase in life expectancy, diseases associated with aging have become the main causes of premature death in developed countries. Cancer, diabetes, cardiovascular diseases (CVD), Alzheimer’s, Parkinson’s, and other neurodegenerative diseases (AD, PD and ND, respectively) are the most common pathologies, which, in best case scenarios, complicate life and very often lead to patient death [1]. In addition, these diseases have highly negative economic consequences for patients, their families and society as a whole. Although both terms, namely, age-associated and aging-associated diseases, are used to define these and some less common pathologies, the latter is more accurate because it is currently clear that the clinical manifestations of these diseases are preceded by long (10-20 years) asymptomatic periods of disease development [2–5]. Substantial efforts to develop methods for the early detection and treatment of aging-associated diseases have led to some promising results, but the overall progress has not been very impressive. There are two major reasons for this relative failure. First, in spite of significant progress in understanding the underlying processes in the development of these diseases, the initiating mechanisms are mostly unclear. In addition, successful treatment of one disease does not lead to significant gains in life span [6–8] because patients die from other pathologies. As a result, the idea that the development of drugs that delay aging will bring more dividends than treatment of particular diseases is becoming increasingly more popular [9–11]. Since all of these diseases are somehow associated with aging, a better understanding of the aging process could clarify the nature of the mechanisms involved in disease initiation and the early stages of development. Recently, the term “Geroscience” was proposed to define the whole realm of aging and aging-related diseases [10].

It is important for a productive hypothesis to include the following: (i) an explanation of the many observations made in a respective area that currently look unrelated to each other; and (ii) a proposal of clear experiments capable of proving or rejecting the hypothesis. In addition, for a hypothesis explaining aging mechanisms, it would be useful to connect the mechanisms of aging with the initiation and development of aging-associated diseases.

There are many hypotheses explaining the nature of aging [12–17], but no uniform theory exists. This paper is not a review, and not all of these hypotheses will be discussed; however, two major concepts explaining aging-associated processes should be mentioned:

1. Aging is a result of cell-autonomous processes, such as accumulation of DNA mutations and aberrant methylations, protein defects, and shortening of telomeres, that can lead to inhibition of cellular proliferation and death of non-dividing terminally differentiated cells (e.g., neurons and cardiomyocytes) or uncontrolled cellular proliferation and tumor development.

Numerous data support this concept, such as those regarding age-related accumulation of various mutations, including oncogenic-inducing changes, aging-associated changes in DNA methylation, shortening of telomeres, and accumulation of defected proteins. These events can lead to cell death, carcinogenic transformation, cellular senescence [13,18], aging of mitochondria and the mitochondrial genome [17,19] and, in turn, manifest in aging of organs and tissues associated with various pathologies. One phenomenon, namely, cell death, clearly plays an important role in aging and aging- associated diseases. The idea of the existence of a genetic cell death program in multicellular eukaryotes, its evolutionary origin and its roles in morphogenesis and regular changes in the cellular populations in both embryogenesis and adult individuals was proposed more than 35 years ago [20]. Very soon after, this hypothesis was confirmed by the discovery of genes whose products were involved in the cell death program [21–24]. In addition, the roles of this program in carcinogenesis and aging were postulated. It was hypothesized that “one of the functions of the cell death program is to eliminate constantly appearing cells with oncogenic features. Hence, for the cell to become malignant two events are necessary, viz. oncogenic mutation and change of the cell death program” [20]. The first proof of this concept was obtained by M. Oren [25,26], who demonstrated for the first time that initiation of the cell death program is an important function of p53 as a tumor suppressor, and p53 mutations are extremely common in different tumor types. Currently, suppression of the cell death program is considered an important step in carcinogenesis. The described hypothesis also considered aging “a pleiotropic effect of cell death program resulting in gradual reduction of the amount of non-dividing cells (most importantly of neurons) damaged or abnormally functioning due to action of various internal and external factors”. At least one study, which demonstrated degenerative processes and early symptoms of aging in mice with hyperactive p53 alleles, supported this idea [27].

Thus, this hypothesis adequately explained many processes in individual cells of multicellular organisms. However, now more than 35 years later with new data obtained, it has become obvious that new ideas are needed to explain various aspects of aging and aging-associated diseases.

2. Aging is a result of a central program that switches on at a specific stage of organismic development [28].

Parabiotic effects of blood or plasma transfusion from young to old animals [28–30], and vice versa [31,32], support this idea.

The following data, which indicate the role of central factors in aging, must be discussed, and the analysis of these data aids in the understanding of the initiating mechanisms of aging and aging-associated diseases.

1. Inverse comorbidity of cancer and Alzheimer’s and other neurodegenerative diseases [33–40].

2. Longer life expectancy for women vs. men and much lower sex-dependent differences, if any, in other mammals [41–46].

3. Higher AD morbidity and lower frequency of many common types of cancer in women vs. men [1,47–49].

4. Higher risk of AD and lower risk of cancer in subjects with Down syndrome [50–54].

5. Increased life span after hypophysectomy [55,56].

6. Rejuvenating effect of blood or plasma transfusion from young donors to elderly recipients in animal models [28–30].

Although various explanations of each of the individual phenomenon listed above have been proposed, the existence of a central regulatory system, as proposed below, will unify and greatly aid in understanding these and other observations.

### HYPOTHESIS

I hypothesize the following: the pituitary, hypothalamus and, perhaps, other endocrine glands, in addition to producing known hormones, also secrete miRNAs, which perform fine tuning of numerous processes, including apoptosis, cellular senescence, mitochondrial changes, autophagy, and insulin, mTOR, Wnt and other signaling pathways. Since the hypothalamus-pituitary gland (PG) axis is regulated by sex hormones, menopause in women and more stepwise changes in circulating sex hormones in men cause sex-dependent changes in miRNA secretion by the PG. These aging-related changes in the spectrum of secreted miRNAs, e.g. increases in pro-apoptotic and decreases in anti-apoptotic miRNAs, although mild and slow, lead to progressive switching from stimulation of developmental processes (proliferation, vascularization, etc.) to their inhibition, thus providing tumor-suppressive effects, and to activation of apoptosis and other degenerative processes [57]. Altogether, when combined with age-related accumulation of various defects in terminally differentiated non-dividing cells, changes in the spectrum of secreted miRNAs result in the manifestation of general aging symptoms, as well as creation of a basis for the initiation and development of various aging-associated diseases*.*

Thus, this hypothesis combines two major concepts of aging: accumulation of molecular damages and central regulation.

### WHY miRNA?

Since the hypothesis described above proposes miRNA as a major player in the new regulatory system, it is necessary to provide a brief introduction to related aspects of miRNA biology.

miRNAs are small molecules (~22 nt) that play an important role in the regulation of target genes by binding to complementary regions of messenger transcripts to repress their translation or to regulate degradation [58,59]. Importantly, more than 2000 miRNAs have been discovered in human cells to date, and many of these miRNAs are enriched in particular organ systems, organs, tissues and cell types [60–63]. Many miRNAs enriched in the brain are differentially expressed in various brain regions, such as the hippocampus, midbrain, frontal cortex, PG, hypothalamus (Table 1), and different cell types, such as neurons and glial cells [64–68].

**Table 1:** miRNAs enriched in hypothalamus, pituitary gland and brain.

miRNA	Hypothalamus	Pituitary gland	Brain enrichment
Let-7a	+	-	+
Let-7b	+	-	-
Let-7c	+	+	+
miR-7	-	+	+
miR-9	-	+	+
miR-16	-	+	-
miR-22	-	+	-
miR-23b	-	+	-
miR-24	-	+	-
miR-26a,b	-	+	-
miR-27b	-	+	-
miR-29a-c	-	+	-
miR-30b,d	-	+	-
miR-92a,b	-	+	+
miR-96	-	+	+
miR-99a,b	-	+	+
miR-103	+	+	+
miR-107	-	+	+
miR-125a,b	+	+	+
miR-126	-	+	-
miR-127	+	+	+
miR-128a	+	+	+
miR-132	+	+	+
miR-134	-	+	+
miR-135a	-	+	+
miR-136	+	+	-
miR-138	+	-	+
miR-141	-	+	-
miR-148a	-	+	-
miR-154	-	+	+
miR-181a-c	-	+	+
miR-182	-	+	+
miR-184	-	+	+
miR-195	-	+	+
miR-197	-	+	+
miR-199b	-	+	-
miR-200a-c	-	+	-
miR-204	-	+	+
miR-212	+	+	+
miR-213	-	+	+
miR-218	-	+	+
miR-323	-	+	+
miR-324	-	+	+
miR-328	-	+	+
miR-329	-	+	+
miR-335	-	+	+
miR-338	+	-	+
miR-339	-	+	-
miR-361	-	+	+
miR-369	-	+	+
miR-370	-	+	+
miR-375	-	+	+
miR-377	-	+	+
miR-379	-	+	+
miR-381	-	+	+
miR-410	-	+	+
miR-411	-	+	+
miR-424	-	+	-
miR-429	-	+	-
miR-432	-	+	+
miR-433	-	+	+
miR-451	+	+	-
miR-487b	-	+	+
miR-491-5p	-	+	+
miR-494	-	+	+
miR-508	-	+	-
miR-514	-	+	-
miR-539	-	+	+
miR-542	-	+	-
miR-628	-	+	+
miR-652	-	+	+
miR-885	-	+	+

miRNAs appear in extracellular space and in bodily fluids (e.g. plasma, serum, urine, saliva, and milk) via mechanisms that are not fully understood. The proposed mechanisms include active secretion in the form of exosomes and miRNA complexes with proteins, blebbing of apoptotic bodies, budding and shedding of microvesicles, etc [69–73]. Due to their small size, secondary structure and location in macromolecular complexes, miRNAs are fairly resistant to RNase activity, and these forms of cell-free miRNA are, therefore, relatively stable in the bloodstream and other bodily fluids. Intracellular concentrations and rates of miRNA secretion can be dramatically affected by physiological and pathological cellular processes [74–76]. It has also been demonstrated in numerous systems that cell-free miRNAs originating from one cell type can be acquired by another cell type (by other cells), resulting in altered expression of proteins due to specific inhibition of messenger RNA (mRNA) targets [77–81]. Thus, the term “miRNA hormones” was proposed [77,79].

Each miRNA can target multiple mRNAs, and one mRNA can be regulated by multiple miRNAs targeting different regions of the 3' untranslated region (UTR). There are several programs that can be used for in silico analysis of the complementarity between miRNA and mRNA; the list of possible targets for some miRNAs frequently includes hundreds of genes [http://mirtarbase.mbc.nctu.edu.tw/php/search.php].

Hence, based on sequence analysis alone, a given miRNA can potentially be involved in numerous pathologies. The same miRNA can function as a tumor suppressor in one cell type and as an oncogene in other cells depending on the spectrum of mRNA targets. In addition, one mRNA can be regulated by numerous miRNAs. Detailed analysis of the available data indicates that significant inhibition of individual miRNA functioning most likely should be caused by several miRNAs [68].

Thus, in spite of all the uncertainties in this field, it is obvious that circulating miRNAs may be good candidates for regulating metabolic processes in distant cells in a hormone-like fashion, and in fact, the regulatory potential of circulating miRNAs has been experimentally demonstrated [77–81].

### WHY the pituitary gland?

The starting point for this hypothesis was the discovery of miRNA biomarkers capable of predicting progression from mild cognitive impairment (MCI) to AD dementia. We investigated the potential use of cell-free miRNAs circulating in the bloodstream for the early detection of AD. Since the early stages of AD are characterized by dysfunction and destruction of synapses that lead to neuronal death in the hippocampus, we hypothesized that these processes should cause additional release of miRNAs enriched in the neurites and synapses of the affected brain area. To compensate for disease-unrelated processes (technical problems, such as isolation of plasma miRNAs or presence of PCR inhibitors, and biological issues, e.g. changes in blood supply and/or blood-brain barrier (BBB) permeability), we also included miRNAs enriched in brain regions that are not affected by AD and several ubiquitous miRNAs to serve as normalization markers. Two families of miRNAs capable of detecting MCI with 87%-96% accuracy were found [82,83]. Since approximately 50% of MCI patients progress to AD dementia, we also looked for biomarkers capable of detecting those patients among MCI subjects. In the first study, four such miRNAs were found, namely, miR-7, miR-125b, miR-16 and miR-451 [84]. Interestingly, the concentrations of these miRNAs in plasma were highly correlated, despite miR-7 and miR-125b being brain-enriched and miR-16 and miR-451 being ubiquitous miRNAs. The only common property that we initially found for these four miRNAs was that they are all highly expressed in the PG [62]. This was the first indication that miRNA secreted from the PG can be associated with AD development. Analysis of potential targets of these and other PG-enriched miRNAs supported the possibility of their involvement in AD development. Bcl-2 and many other apoptosis-related genes are among the predicted targets, and subsequently, the involvement of these miRNAs in the regulation of apoptosis was experimentally proved [85–91]. In addition, among the potential targets of these miRNAs are genes involved in various pathways associated with aging-related diseases, such as insulin, TOR, Wnt signaling, autophagy and other pathways. These miRNAs, the abnormally high concentrations of which in plasma predict MCI progression to AD dementia, serve as tumor suppressors in various organs. Another fact implicating the PG as a source of such regulation is the above-mentioned anti-aging effect and extension of the lifespan caused by hypophysectomy in adult animals [55,56]. In addition, it was previously demonstrated that dwarf mice have longer lifespans and that df/df/APP/PS1 hybrid mice, a cross between dwarf mice and double transgenic mice expressing human mutant amyloid precursor protein (APP) and presenilin-1 (PS1), have reduced Aβ plaque deposition and less Aβ 1-40 and Aβ 1-42 concentrations in the brain [92]. Notably, the age effect on plasma concentrations of PG-enriched miRNAs (e.g., miR-127-5p, miR-154, miR-369, miR-381, miR-410, and miR-411) is 10-20 times lower in dwarf mice than in normal controls [93]. Recently, we performed our own study of sex- and aging-dependent changes in the spectrum of brain-enriched miRNAs in human plasma and found, among other observations, sex-dependent changes in the spectrum of miRNA hormones secreted by the PG during aging [94]. It should be mentioned that, although the mechanisms by which miRNAs cross the BBB are not well understood, the presence of brain-specific and brain-enriched miRNAs in human and animal plasma/serum and other bodily fluids, supported by a growing body of data [95–99], indicates that these miRNAs are able to cross the BBB. miRNA-containing exosomes and other microvesicles, as well as complexes with proteins and lipoproteins, are being investigated as potential carriers of miRNAs across the body barriers. Some data indicate that miRNAs are involved in regulation of the cerebrovascular network of the brain and may affect BBB disruption [100–103] leading to vascular cognitive impairment. Thus, while it is clear that miRNA hormones secreted by the PG can affect neurons, glial and vascular cells in the brain, the mechanisms by which they appear in the bloodstream still need to be elucidated. Further, the proposed hypothesis does not exclude a role of locally synthesized miRNAs or of miRNAs expressed in different glands or in the cells of the immune system. Although in the present paper the hypothesis is mainly explained on the basis of central regulation of cell death, miRNA hormones may be involved in regulation of other aging-related processes. Cell death was chosen as an example here because the role of cell death in aging and aging-associated diseases, as well as the mechanisms of cell death and involvement of miRNAs in the regulation of apoptosis, have been investigated to date in greater detail.

There are other observations supporting the above hypothesis: (i) androgen-deprivation therapy in the treatment of prostate cancer is associated with an increased risk of dementia [104]; (ii) women with surgically premature menopause have an increased risk of both MCI and AD [105]; (iii) injection of PG extract in growth hormone treatment led to Aβ deposition [106,107]; and (iv) finally, changes in the spectrum of miRNAs secreted by the hypothalamus and the role of these miRNAs in aging has recently been demonstrated [108], results that were in good agreement with the proposed hypothesis since the role of the hypothalamus in the regulation of hormone secretion by the PG is a well-known phenomenon. Most likely, this phenomenon is similar to changes in the secretion of other PG hormones due to the decrease in the concentrations of sex hormones by the end of the female reproductive period, which is important for preventing the accumulation of mutations in germ cells. Importantly, females in other mammalian species, including non-human primates, do not possess the menopause characteristic of women in mid-life [109]. In addition, aged monkeys and apes (as well as dogs) can accumulate large quantities of Aβ but remain without a dementia-like disorder [110].

### Explanation of various aging-associated processes in the context of the proposed hypothesis

In this chapter, I will briefly summarize how the proposed hypothesis explains the phenomena outlined in the Introduction.

1. Inverse comorbidity of cancer and Alzheimer’s and other neurodegenerative diseases. Prospective and retrospective studies performed in different countries have convincingly demonstrated that the chances of developing cancer are significantly lower than average for patients with AD and other neurodegenerative diseases. Similarly, cancer survivors have lower chances of developing AD [33–40]. There are two miRNA-associated factors that can explain why subjects with AD have a lower chance of developing cancer, and subjects who survive cancer have a lower probability of developing AD. First, if the PG secretes more pro-apoptotic miRNAs (e.g. Bcl-2-inhibiting miRNAs), this will decrease the chance of developing cancer but increase the chances of developing neuro- and other degenerative diseases, and vice versa; higher levels of anti-apoptotic miRNAs stimulate cancer development but decrease the chance of developing AD. Of course, the same is true for miRNAs that regulate other cancer- and degeneration-related pathways. Second, it is interesting that many synapse/dendrite-enriched miRNAs that are released in the early stages of neurodegenerative diseases due to neurite dysfunction and destruction and then circulate in the bloodstream are pro-apoptotic, which decreases the chance of developing cancer. On the other hand, tumor cells secrete anti-apoptotic miRNAs that can inhibit degenerative processes, though the ability of these miRNAs to reach the brain is questionable.

2. Higher AD morbidity and lower frequency of many common types of cancer in women vs. men. Two-thirds of Americans living with AD dementia are women, and neither their longer lifespans nor differences in lifestyle compared to men can explain these numbers. It has been suggested that the higher frequency of female AD morbidity is caused by increased chances of AD initiation earlier in life due to menopause [111,112], although the mechanisms underlying this phenomenon are not clear. The proposed hypothesis explains these sex differences by changes in the spectrum of secreted PG miRNA hormones from pro-developmental to anti-carcinogenic, changes that are associated with decreased levels of estrogen. This switch decreases the chances of carcinogenesis and increases the chances of neurodegenerative processes. Due to menopause, all of these processes start in females about 10 years earlier than in males. The estrogen dependence of many PG-enriched miRNAs [113], as well as sex-dependent differences in their plasma concentrations in the period of 46-65 years of age [94], has been recently demonstrated.

3. Higher risk of early AD and lower risk of cancer in subjects with Down syndrome. Since menopause in females and the decrease in sex hormone production in male subjects with Down syndrome occur much earlier than in healthy subjects, the switch in the spectrum of PG-secreted miRNAs described in the previous paragraph decreases the chances of carcinogenesis and increases the chances of neurodegenerative processes.

4. Longer life expectancy in women vs. men and much lower sex-dependent differences, if any, in other mammalian species. Again, this phenomenon can be explained by the earlier switch in the spectrum of PG-secreted miRNA hormones in women than in men. This phenomenon results in more effective elimination of cells with dangerous mutations and other abnormalities and decreased chances of cancer due to menopause. Since other mammalian species do not undergo menopause, there are no sex differences throughout the lifespan.

5. Hypophysectomy increases lifespans. Many labs using different animals have demonstrated that hypophysectomies performed after organism development increase lifespans. Dwarf mice, which have a hypofunctional PG, have less cancer and longer lifespans. Victoria et al. [93] demonstrated significantly different spectrums of miRNAs circulating in the plasma of normal and Ames dwarf mice. Many of these miRNAs are enriched in the PG. These data indicated the roles of the PG and secreted miRNAs in normal aging and lifespan; however, much more detailed studies are needed to explain the metabolic changes involved.

6. Parabiotic rejuvenating effect of blood or plasma transfusion from young donors to elderly recipients in animal models. It is quite possible that the effect of plasma transfusion is at least partially caused by circulating miRNAs. The inhibitory effect of plasma-heating denaturation [26], often interpreted as an indication of the protein nature of active parabiotic compounds, does not exclude miRNA participation since after such treatment, the miRNAs are degraded in the circulation by RNases.

Currently, the proposal and discussion of specific mechanisms of miRNA hormone actions based on their potential targets does not make much sense since, as discussed above, each miRNA can regulate numerous mRNA targets, and each mRNA can be regulated by many miRNAs. Thus, the effects of miRNA hormones and of their spectrum switch due to the decrease in sex hormone levels can be different in various tissues, being determined by gene expression profiles. At the same time, if several miRNA hormones that affect the same process are changed in one direction (e.g. miR-7, miR-16, miR-125b and miR-451a, all of which inhibit bcl-2 expression), one can expect a resulting modulation of apoptosis.

### Experimental tests of the hypothesis

Of course, as with any hypothesis, this one, in addition to explaining numerous observations, needs to be proven experimentally. The following studies could be useful to prove or reject the proposed hypothesis:

1. Study of age-dependent changes in circulating miRNA (e.g., pro- and anti-apoptotic) concentrations.

2. Detailed analysis of age- and sex-dependent miRNA expression in human and animal PGs and the correlation of this expression with sex hormone levels.

3. miRNA secreted in vitro by the PGs of humans and animals of different ages.

4. Analysis of the forms (exosomes and other vesicles, complexes with proteins and lipids) of PG-secreted miRNAs, their correlations with the frequency of aging-associated diseases and the effects on apoptosis in different cell types.

5. Retrospective studies of circulating PG-enriched miRNAs in pre-AD and pre-cancer subjects.

6. Parabiotic effects of different fractions of circulating miRNAs.

7. Cancer frequency and miRNA levels in plasma after hypophysectomy, low-calorie diets, etc.

8. Testing the effects of castration or sex hormone inhibition in animal AD models.

Many more experiments can be proposed, including modeling with sex hormone injections and analysis of in vivo changes in miRNA expression in the PG or in circulation.

## BRIEF SUMMARY

The objective of this paper is to stimulate the exchange of ideas and research in the area of aging regulation by miRNA hormones secreted by the hypothalamus-PG axis. The proposed concept combines local and central mechanisms of aging and its associated processes and is consistent with many observations that are currently difficult to explain. Apoptosis was selected as an example since it is one of the most investigated phenomena in this area, and miRNAs involved in the regulation of apoptosis have been investigated relatively well. Clearly, this concept can be applied to other processes in the initiation and realization of aging, such as cellular senescence [13,18], aging of the mitochondrial genome and of mitochondria in general [17,19], DNA methylation [114], and other processes. Similarly, neurodegenerative diseases and carcinogenesis are discussed as two examples of aging-associated pathologies because, on the one hand, there are numerous observations both tying these factors to aging and, on the other hand, contraposing them to each other. In addition, apoptosis is involved in the regulation of both pathologies, thereby simplifying the presentation of the hypothesis. Other pathological processes associated with aging may be regulated by miRNA hormones and hopefully will be investigated and discussed in the future. Some of these processes, such as cerebrovascular injuries leading to BBB dysfunction and changes in blood supply, are directly involved in the processes underlying neurodegeneration [100–103]. In addition, the proposed hypothesis does not exclude alternative mechanisms contributing to aging, such as telomere shortening, DNA methylation, mitochondrial damages, effects of other hormones, etc.

The proposed hypothesis introduces the idea that sex hormone-dependent changes in the spectrum of PG/hypothalamus-secreted miRNA hormones increase the chances of apoptosis caused by the accumulation of age-related molecular defects, and thus, this hypothesis combines two major concepts of aging: the accumulation of molecular damages and central regulation. If this hypothesis is proven by experimental testing, it will have numerous practical applications, such as the following: (i) aging modification via application of sex hormones under the control of circulating miRNA hormones; (ii) aging modification via separate delivery of respective miRNA hormones into the brain and body blood circulation, which can be even more effective after preliminary DNA sequencing to evaluate a subject’s predisposition to various pathologies; (iii) treatment of AD with delivery of anti-apoptotic miRNA hormones to the brain with no cancer activation; and (iv) creation of better animal AD and other aging-associated disease models by introducing artificial menopause.
